# Posttraumatic stress disorder, trauma, and accelerated biological aging among post-9/11 veterans

**DOI:** 10.1038/s41398-023-02704-y

**Published:** 2024-01-06

**Authors:** Kyle J. Bourassa, Melanie E. Garrett, Avshalom Caspi, Michelle Dennis, Katherine S. Hall, Terrie E. Moffitt, Gregory A. Taylor, VA Mid Atlantic MIRECC Workgroup, Jean C. Beckham, Patrick S. Calhoun, Eric Dedert, Eric B. Elbogen, Robin A. Hurley, Jason D. Kilts, Nathan A. Kimbrel, Angela Kirby, Sarah L. Martindale, Christine E. Marx, Scott D. McDonald, Scott D. Moore, Rajendra A. Morey, Jennifer C. Naylor, Jared A. Rowland, Robert Shura, Cindy Swinkels, Elizabeth E. Van Voorhees, H. Ryan Wagner, Anna T. Magnante, Victoria L. O’Connor, Pallavi Aurora, Brandy S. Martinez, Tate F. Halverson, Allison E. Ashley-Koch, Jean C. Beckham, Nathan A. Kimbrel

**Affiliations:** 1 Geriatric Research, Education, and Clinical Center, Durham VA Health Care System, Durham, USA; 2 VA Mid-Atlantic Mental Illness Research, Education and Clinical Center, Durham VA Health Care System, Durham, USA; 3 Center for the Study of Aging and Human Development, Duke University Medical Center, Durham, USA; 4 Duke Molecular Physiology Institute, Duke University, Durham, USA; 5 Department of Psychiatry and Behavioral Sciences, Duke University School of Medicine, Durham, US; 6 Department of Psychology and Neuroscience, Duke University, Durham, USA; 7 Institute of Psychiatry, Psychology and Neuroscience, King’s College London, London, UK; 8 Center for the Study of Population Health & Aging, Duke University Population Research Institute, Durham, USA; 9 Department of Medicine, Division of Geriatrics, Duke University, Durham, USA; 10 Department of Integrative Immunobiology, Duke University Medical Center, Durham, USA; 11 VA Health Services Research and Development Center of Innovation to Accelerate Discovery and Practice Transformation, Durham VA Health Care System, Durham, USA; 12 SBYVAMC, Wake Forest University, Winston-Salem, USA; 13 SBYVAMC, Wake Forest University School of Medicine, Winston-Salem, USA; 14 RVAMC, Virginia Commonwealth Univ, Richmond, VA USA

**Keywords:** Psychiatric disorders, Human behaviour, Clinical genetics

## Abstract

People who experience trauma and develop posttraumatic stress disorder (PTSD) are at increased risk for poor health. One mechanism that could explain this risk is accelerated biological aging, which is associated with the accumulation of chronic diseases, disability, and premature mortality. Using data from 2309 post-9/11 United States military veterans who participated in the VISN 6 MIRECC’s Post-Deployment Mental Health Study, we tested whether PTSD and trauma exposure were associated with accelerated rate of biological aging, assessed using a validated DNA methylation (DNAm) measure of epigenetic aging—DunedinPACE. Veterans with current PTSD were aging faster than those who did not have current PTSD, β = 0.18, 95% CI [0.11, 0.27], *p* < .001. This effect represented an additional 0.4 months of biological aging each year. Veterans were also aging faster if they reported more PTSD symptoms, β = 0.13, 95% CI [0.09, 0.16], *p* < 0.001, or higher levels of trauma exposure, β = 0.09, 95% CI [0.05, 0.13], *p* < 0.001. Notably, veterans with past PTSD were aging more slowly than those with current PTSD, β = -0.21, 95% CI [-0.35, -0.07], *p* = .003. All reported results accounted for age, gender, self-reported race/ethnicity, and education, and remained when controlling for smoking. Our findings suggest that an accelerated rate of biological aging could help explain how PTSD contributes to poor health and highlights the potential benefits of providing efficacious treatment to populations at increased risk of trauma and PTSD.

## Introduction

Posttraumatic stress disorder (PTSD) is a common [1, 2] and costly [3, 4] mental health disorder that is linked to poorer health [5–7], including greater risk of chronic disease [5, 6], disability [8], and premature death [7]. Despite well-established epidemiological evidence linking PTSD to poor health, it is unclear what explains these health consequences [9–11]. It is critical to determine how PTSD might lead to poorer health to facilitate future interventions that might mitigate the health consequences of PTSD [10]. This is particularly true for populations at greater risk of developing PTSD, such as first responders and military veterans [12–14], who might benefit the most from such interventions.

A number of plausible physiological mechanisms have been theorized to explain the causal pathway from PTSD to poor health. Prior empirical work has shown PTSD can disrupt immune, endocrine, and circulatory system function [9, 15–18], as well as psychosocial mechanisms linked to health, including reduced social support [19, 20] and unhealthy behaviors [21]. Given the breadth of these findings, it is likely that the poor health observed among those with PTSD arise from multiple causes spanning psychosocial, behavioral, and physiological dysregulation. With many plausible mechanistic pathways, there is a need to establish health-relevant biomarkers that can link PTSD to poor health and act as proximal outcomes for interventions studies aiming to reduce the health consequences associated with PTSD.

Accelerated biological aging is a novel mechanism that might help explain how PTSD could result in poor health that manifests across multiple physiological systems [22, 23] and risk for several chronic diseases. Biological aging represents the rate at which people’s physiological function declines, which differs among people of the same chronological age. People with accelerated biological aging are theorized to be at risk of poor health across multiple organ systems [24], making assessments of biological aging particularly useful as surrogate clinical outcomes relevant to health [25, 26]. New advances in assessing biological aging using epigenetic DNA methylation measures (DNAm) has enabled more efficient and timely measurement of biological aging [27, 28], particularly third-generation epigenetic measures such as DunedinPACE [27] that have been trained to predict previously-validated measures of biological aging [28]. Epigenetic aging measures are promising as biomarkers that could serve as outcome for human intervention trials with relevance to health and longevity [25, 29].

Empirical work has begun to support the theory that people with PTSD might evidence poorer health due to more rapid aging following the experience of trauma [30, 31]. Recent studies have found PTSD is associated with epigenetic measures of biological aging in both civilian [32] and military veteran populations [33–35]. However, prior studies are limited by smaller samples and often include primarily non-Hispanic White participants. Prior studies are also primarily focused on comparing people with current PTSD to people without current PTSD. Physiological dysregulation associated with PTSD in the cardiovascular system can be altered through PTSD treatment [18, 36], which makes it equally interesting to study whether people with PTSD in the past that is currently in remission have faster or slower aging. Relatively fewer studies have examined whether a history of PTSD is associated with different rates of aging, which could help provide initial evidence as to the reversibility of accelerated aging associations to support future interventions. There is a pressing need to test the association of trauma and PTSD with accelerated aging in larger and more diverse samples to support intervention efforts to improve health for people who have experienced trauma and PTSD.

### Present study

The current study included 2309 participants from the Post-Deployment Mental Health Study (PDMH; 37)—a cohort of U.S. veterans deployed following September 11, 2001. Participants provided blood samples used to derive DNA methylation (DNAm) scores and were assessed for trauma and PTSD. We tested the association between trauma, PTSD, and epigenetic biological aging using PDMH data. Biological aging was assessed using DunedinPACE, a validated third-generation DNAm measure of aging trained directly on longitudinal trajectories of age-related biomarkers [27, 28, 37, 38]. We hypothesized that veterans with current PTSD would show accelerated aging compared to veterans without PTSD. We also expected that veterans with higher PTSD symptoms and more trauma exposure would evidence accelerated aging. We also tested whether PTSD (diagnostic status and symptoms) and trauma burden were unique predictors of biological aging when included in the same model. Finally, we examined the history of PTSD and accelerated aging—we hypothesized that veterans with past PTSD would be aging at a rate more similar to veterans without a history of PTSD as compared to those with current PTSD.

## Methods

### Participants and study design

Participants were members of the PDMH [39], multi-site study of US Afghanistan and Iraq era veterans. The PDMH study protocol was approved by the Durham VAMC Institutional Review Board and all participants consented to participate. The VA Mid-Atlantic (VISN 6) MIRECC began enrolling participants in 2005 as a regional cohort data repository to facilitate mental health research focused on the millions of the troops returning from post-9/11 deployment. This cohort has faced substantial mental health challenges—over half of the veterans in the PDMH sample who have sought VA care have at least one mental health diagnosis and they have increasingly utilized VA health care as they enter midlife. The cohort is notably diverse—over 20% of the sample identified as women and approximately half the participants identified as African-American/Black. The current study included participants who had DNA methylation data available, were assessed for PTSD, and comprised the two major self-reported racial/ethnic groups (non-Hispanic Black and non-Hispanic White), resulting in a final sample of 2 309 veterans. Demographics and other characteristics of the sample are described in the Results.

### Measures

#### Biological aging

Biological aging was assessed using a well-validated epigenetic measure of aging, DunedinPACE, applied to DNAm data from the PDMH cohort [40] as part of the larger PDMH survey. As described in detail elsewhere [40], whole blood samples were collected via venipuncture. In total, 2 444 samples with sufficient DNA yield and quality were analyzed for methylation CpG sites using either the Infinium HumanMethylation450 or MethylationEPIC Beadchip (Illumina Inc, San Diego, CA). Internal replicates were included and checked for consistency using single nucleotide polymorphisms (SNPs) on each array. Quality control (QC) was performed using the minfi [41] and ChAMP [42] R packages. Samples were excluded if average fluorescence signal intensity was below 2000 arbitrary units or <50% of the mean intensity of all samples, >10% of probes were not detectable (*p*-value > 0.001), if a sex mismatch was detected, or if the sample was deemed an outlier on principal component analysis plots. In total, 134 samples were removed due to QC, producing 2310 samples. Probe QC and data normalization was performed within each batch using the R package wateRmelon [43]. Probes not detected (detection *p*-value > 0.001) in >10% of samples and those hybridizing to multiple locations in the genome were removed. Raw beta values were normalized using the dasen approach [43] and batch and chip adjustments were accomplished using ComBat in the R package sva [44]. Methylation values reflected the resulting normalized and adjusted beta values. The DunedinPACE algorithm [38] was applied to these values using existing code and produced a biological aging score for each participant. DunedinPACE is currently the only epigenetic measure of aging trained on longitudinal trajectories of age-related biomarkers that assess the rate of biological aging, specifically the Pace of Aging [45, 46], and uses CpG probes that are reliable across methylation chips [47]. Resulting values for each veteran’s epigenetic aging scores represents years of biological aging per chronological year (i.e. expected aging), with higher scores representing faster aging. Additional description of DunedinPACE and the original biomarker-assessed measure of biological aging, the Pace of Aging, is included in Supplemental Text 1. Additional models testing the main study findings while controlling for proportion of cell counts are also presented in Supplemental Analyses 1 and Supplemental Table 1. The primary study results replicated in these models.

#### Posttraumatic stress disorder (PTSD)

PTSD was assessed two ways, first using diagnostic criteria in a clinical interview and second using a self-report measure of PTSD symptoms. PTSD diagnostic status was assessed using the Diagnostic Interview Schedule [48] according to the current versions of *DSM-IV*. Diagnostic status first assessed whether participants met criteria for current PTSD. If participants had a criterion A trauma but did not meet criteria for current PTSD, they were then assessed for whether they met criteria in the past. In total, 32.4% of participants assessed had current PTSD, with an additional 9.8% meeting criteria for past PTSD. Self-reported PTSD symptoms were assessed using the Davidson Trauma Scale (DTS) [49], a 17-item self-report measures assessing PTSD symptoms. Items use a 5-point Likert-scale for both frequency and intensity of symptoms over the past week with higher scores corresponding to greater PTSD symptoms. The DTS has been previously validated among post-9/11 veterans [50]. Total DTS scores were used to represent PTSD symptoms, with higher scores representing more symptoms.

To provide the most inclusive measure of PTSD and replace a subset of missing interview data (*n* = 102, 4.4% of the total sample), we combined the diagnostic interview and DTS results to derive our measure of current PTSD. Participants were coded as having current PTSD if they met interviewer-rated diagnostic criteria for PTSD or had a DTS score of 35 or above, which is a reliable and valid clinical cutoff with specificity of 0.95 and sensitivity of 0.91 [50]. This resulted in an additional 454 participants meeting criteria for current PTSD. Primary study results using only the diagnostic interview PTSD diagnoses or DTS clinical cutoff are included in the supplement. As shown in Supplemental Table 2, the primary study results replicated when assessing PTSD diagnostic status using either interviewer-assessed or self-report-assessed diagnostic status independently.

#### Trauma exposure

Trauma exposure was assessed using the Traumatic Life Events Questionnaire (TLEQ; [51]. The TLEQ is a self-report measure that assesses whether participants experienced 22 categories of potentially traumatic events across the lifespan. The number of categories of potentially traumatic events participants experienced were summed to create an index of traumatic event burden across the lifespan, with higher scores representing relatively more traumatic experiences.

#### Study covariates

Participants self-reported their age, gender, race and ethnicity, years of education, and smoking status. Smoking was assessed using a three-point scale ranging from never smoked to past smoking and to current smoking.

### Data analysis

We used a series of multiple regression models to test the associations between PTSD, trauma, and epigenetic biological aging. We first tested the association between current PTSD status and biological aging assessed by DunedinPACE. Second, we tested the association between PTSD symptoms and DunedinPACE. Third, we tested the association between level of trauma exposure and DunedinPACE. Finally, we tested the association between past PTSD and DunedinPACE and compared this group’s biological aging to that of participants who never had PTSD and those with current PTSD. For each association of interest, we specified four models with an increasing number of covariates. Our first model assessed the bivariate age-adjusted association, which regressed DunedinPACE on chronological age and the predictor of interest. The second model then controlled for additional demographic covariates (gender, race/ethnicity, and years of education) and type of methylation chip, and the third model accounted for smoking status (due to well-established effects of smoking on DNAm). We also conducted three secondary analyses to contextualize our main findings, specifically by assessing the association of DunedinPACE with PTSD and trauma in the same models, stratifying our main results by self-reported race/ethnicity and gender, and by using a smoking methylation score as a covariate instead of self-reported smoking status. All models were run in MPLUS version 8.3 [52] using full maximum likelihood estimation to account for missing data (MPLUS code can be requested from the corresponding author). All βs reported reflect standardized effect sizes, whereas *Bs* reflect unstandardized values.

## Results

Of the 2309 veterans included in the current study, 491 reported they were women (21.3%) and 1,109 (48.0%) reported their race/ethnicity as non-Hispanic Black. On average, the sample was 37.4 years old (*SD* = 10.1 years) and had 13.6 years of education (*SD* = 3.6). In total, 1,168 participants (50.6%) met criteria for current PTSD. The majority of participants (*n* = 1 193, 51.9%) reported having never smoked, with 527 reporting past smoking (22.9%), and 579 reporting current smoking (25.2%). The veterans’ average rate of biological aging in the sample assessed by DunedinPACE was 1.07 (*SD* = 0.11) and ranged from 0.75 to 1.57. DunedinPACE aging scores were correlated with chronological age, β = 0.23, 95% CI [0.19, 0.27], *p* < .001. Participants reported experiencing 7.1 categories of trauma (*SD* = 3.5) in their lifetime on average.

### Current PTSD and accelerated aging

**Fig. 1:**
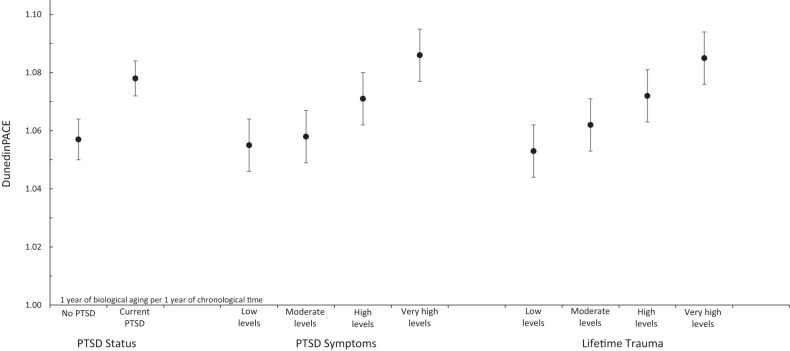
Biological aging scores—measured by DunedinPACE and scaled to years of biological aging for each year of chronological aging—based on current PTSD status, PTSD symptoms, and lifetime trauma burden. PTSD status included participants with (*n* = 1,168) and without current PTSD (*n* = 1,141). Veterans with PTSD were aging faster, β = 0.18, 95% CI [0.11, 0.27], *p* < .001. Categories for PTSD symptoms and lifetime trauma burden were created using quartile splits across the full sample (*n* = 2,309) and are for illustrative purposes only—analyses using PTSD symptoms and trauma burden were measured continuously. Veterans with more PTSD symptoms and more trauma were aging faster, β = 0.13, 95% CI [0.09, 0.16], *p* < .001, and β = 0.09, 95% CI [0.05, 0.13], *p* < 0.001, respectively. Error bars represent 95% confidence intervals.

Veterans with current PTSD were aging faster biologically compared to veterans without PTSD, β = 0.23, 95% CI [0.15, 0.31], *p* < 0.00 (Table 1, Fig. 1). Veterans with current PTSD continued to show faster DunedinPACE when controlling for demographic covariates, β = 0.22, 95% CI [0.14, 0.30], *p* < 0.001, as well as when accounting for smoking status, β = 0.14, 95% CI [0.06, 0.22], *p* < 0.001. The size of the unstandardized effect controlling for demographic covariates (*B* = 0.03) was equivalent to 0.4 months of additional aging per year. Said differently, veterans with current PTSD were biologically aging 0.4 months more per chronological year on this measure compared to veterans without PTSD on average.

**Table 1:** Association of DunedinPACE with PTSD and Trauma among post-9/11 veterans.

	Age-adjusted Bivariate		Adding demographics		Adding smoking status	
*N* = 2309	β	95% CI	β	95% CI	β	95% CI
**Current PTSD**	0.23**	[0.15, 0.31]	0.22**	[0.14, 0.30]	0.14**	[0.06, 0.22]
Age	0.25**	[0.21, 0.28]	0.24**	[0.20, 0.28]	0.27**	[0.23, 0.31]
Gender			0.31**	[0.22, 0.41]	0.34**	[0.25, 0.43]
Race/ethnicity			−0.29**	[−0.36, −0.21]	−0.37**	[−0.44, −0.29]
Education			−0.07**	[−0.11, −0.03]	−0.04*	[−0.08, −0.00]
Methylation chip			−0.13**	[−0.23, −0.03]	−0.15**	[−0.24, −0.06]
Smoking Status					0.33**	[0.29, 0.36]
**PTSD symptoms**	0.13**	[0.09, 0.17]	0.13**	[0.09, 0.16]	0.08**	[0.04, 0.11]
Age	0.25**	[0.21, 0.28]	0.25**	[0.21, 0.29]	0.27**	[0.23, 0.31]
Gender			0.31**	[0.22, 0.41]	0.34**	[0.25, 0.43]
Race/ethnicity			−0.28**	[−0.35, −0.20]	−0.36**	[−0.43, −0.28]
Education			−0.06**	[−0.10, −0.02]	−0.04	[−0.07, 0.00]
Methylation chip			−0.13**	[−0.23, −0.03]	−0.15**	[−0.24, −0.06]
Smoking Status					0.32**	[0.29, 0.36]
**Lifetime trauma burden**	0.09**	[0.05, 0.13]	0.09**	[0.05, 0.13]	0.05**	[0.01, 0.09]
Age	0.23**	[0.19, 0.27]	0.23**	[0.19, 0.27]	0.26**	[0.23, 0.30]
Gender			0.28**	[0.19, 0.38]	0.32**	[0.23, 0.41]
Race/ethnicity			−0.29**	[−0.36, −0.21]	−0.37**	[−0.44, −0.29]
Education			−0.08**	[−0.12, −0.04]	−0.05*	[−0.08, −0.01]
Methylation chip			−0.16**	[−0.26, −0.06]	−0.17**	[−0.26, −0.08]
Smoking Status					0.33**	[0.29, 0.36]

*Note*: Current PTSD indicates participants with current PTSD or no current PTSD, PTSD symptoms measures self-reported PTSD symptoms, and lifetime trauma burden assesses count of trauma categories experienced across the lifespan. Each model adds more covariates to the model. Current PTSD is coded 0 = no PTSD, 1 = current PTSD; gender is coded 0 = men, 1 = women; Race/ethnicity is coded 0 = non-Hispanic Black, 1 = non-Hispanic White; methylation chip is coded 0 = Infinium HumanMethylation450 BeadChip, 1 = Infinium MethylationEPIC BeadChip, smoking status is coded 0 = never smoked, 1 = past smoking, 2 = current smoking. *CI* confidence interval.

*p < 0.05. **p < 0.01.

### PTSD symptoms and accelerated aging

Veterans who reported more PTSD symptoms also had faster DunedinPACE compared to veterans reporting fewer PTSD symptoms, β = 0.13, 95% CI [0.09, 0.17], *p* < 0.001 (Table 1, Fig. 1). Veterans with higher levels of PTSD symptoms continued to show faster DunedinPACE when controlling for demographic covariates, β = 0.13, 95% CI [0.09, 0.16], *p* < 0.001, and when accounting for smoking status, β = 0.08, 95% CI [0.04, 0.11], *p* < 0.001.

### Trauma burden and accelerated aging

Veterans who reported more trauma exposure showed faster DunedinPACE compared to veterans with less trauma exposure, β = 0.09, 95% CI [0.05, 0.13], *p* < 0.001 (Table 1, Fig. 1). Veterans with more trauma exposure continued to show faster DunedinPACE when controlling for demographic covariates, β = 0.09, 95% CI [0.05, 0.13], *p* < 0.001, and when accounting for smoking status, β = 0.05, 95% CI [0.01, 0.09], *p* = 0.007.

### Past PTSD and accelerated aging

In the subset of veterans (*n* = 1221) who were diagnosed with either current (*n* = 1005) or past PTSD (*n* = 216) using interview-rated DSM-IV criteria, veterans with past PTSD evidenced slower DunedinPACE than those with current PTSD, β = -0.18, 95% CI [-0.32, -0.04], *p* = .009. This association remained when controlling for demographic covariates, β = -0.21, 95% CI [-0.35, -0.07], *p* = .003, as well as when accounting for smoking status, β = -0.18, 95% CI [-0.32, -0.04], *p* = .006. Descriptively, the average DunedinPACE aging score for the veterans with past PTSD (1.059) was more similar to the aging score of veterans without PTSD (1.058) than the score of veterans with current PTSD (1.080), see Fig. 1.

### Secondary analysis: assessing trauma and PTSD in the same models

**Fig. 2:**
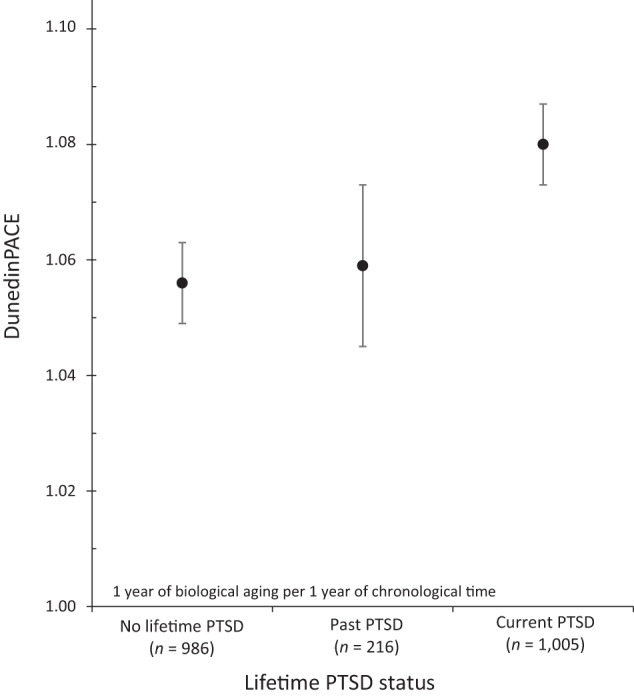
Biological aging scores for veterans with no lifetime PTSD, past PTSD, and current PTSD. Biological aging is measured by DunedinPACE and scaled to years of biological aging for each year of chronological aging. The total *N* for this figure is 2,207, which excludes 102 (4.4% of the study sample) who did not have interviewer-rated data, which included past PTSD diagnosis. Veterans with past PTSD were aging significantly slower than veterans with current PTSD, β = -0.18, 95% CI [-0.32, -0.04], *p* = .014. Error bars represent 95% confidence intervals.

We ran additional models assessing the additive association of trauma exposure and PTSD with DunedinPACE in the same models. Both current PTSD status, β = 0.19, 95% CI [0.11, 0.28], *p* < .001, and trauma burden were significantly associated with DunedinPACE when included in the same model, β = 0.06, 95% CI [0.02, 0.10], *p* = .007. These associations remained when controlling for demographic covariates. PTSD status remained associated with DunedinPACE when also accounting for smoking status, β = 0.12, 95% CI [0.04, 0.20], *p* = .003, however the association for trauma burden was no longer significant, β = 0.03, 95% CI [-0.01, 0.07], *p* = .126. This pattern of associations replicated when using PTSD symptoms and trauma in the same model, rather than PTSD status (Fig. 2).

### Secondary analysis: stratifying by gender and self-reported race/ethnicity

We conducted additional analyses to examine our models while stratifying by self-reported race/ethnicity (non-Hispanic Black and non-Hispanic White) and gender (men and women). Descriptively, the associations of PTSD diagnostic status, PTSD symptoms, and trauma burden with DunedinPACE were stronger among non-Hispanic White veterans compared to non-Hispanic Black veterans. Associations between PTSD and DunedinPACE were non-significant in some cases among the non-Hispanic Black veterans; however, all estimates of the associations of PTSD and trauma with DunedinPACE were in the positive direction. The associations of PTSD diagnostic status and PTSD symptoms with DunedinPACE were largely similar between men and women, whereas the association between trauma burden and DunedinPACE was descriptively, but not statistically, stronger among women. Full results for the models are presented in Supplemental Table 3.

### Secondary analyses: controlling for smoking history as assessed by methylation

Although our primary findings replicated while controlling for self-reported smoking status, it is possible that veterans’ history of cigarette use was either incorrectly reported or did not account for prior/current levels of use. As a result, we conducted additional secondary analyses examining our primary findings when controlling for a methylation measure of smoking history [53, 54]. As shown in Supplemental Analysis 2, this smoking methylation score correlated with self-report smoking status (*r* = .56, *p* < .001) and all primary study results replicated while controlling for the methylation measure of smoking history.

## Discussion

In the current study, we investigated PTSD, trauma, and epigenetic biological aging in a cohort of veterans from post-9/11 military deployments (*N* = 2 309). We found that participants with current PTSD, higher levels of PTSD symptoms, and higher levels of trauma exposure had accelerated rates of biological aging, assessed by a third-generation epigenetic measure of aging [27]. Veterans who had a history of PTSD but did not meet current criteria were aging at a rate that was more similar to veterans without a history of PTSD compared to those with current PTSD.

These findings have theoretical and clinical implications. Theoretically, this study provides additional support for the hypothesized association between PTSD and accelerated biological aging [29, 31, 32] and suggests that biological aging may be a physiological mechanism that helps explain how PTSD contributes to poorer health [30–32]. The observed differences in DunedinPACE scores reflect a difference in the expected rate of aging people might experience over the next months and/or years. The rate at which individuals are aging can be contrasted with other epigenetic measure of biological age, such as epigenetic clocks [28] that aim to estimate biological age at a given point in time. This may make measures of the rate of aging, such as DunedinPACE, more appropriate to assess changes in the rate of aging that might occur due to treatable mental health conditions, such as PTSD. This work will help support efforts to outline the casual pathways that might link PTSD to accelerated aging more fully. For example, it is possible that PTSD symptoms or the experience of trauma do not directly accelerate aging, but instead do so through health-relevant psychosocial sequelae of PTSD, such as health behaviors [9–11] or social isolation [19, 20]. Many plausible direct and indirect causal pathways could explain these associations [9–11] and future study is needed to better understand the causal pathways linking PTSD to aging and health. Different explanations would implicate different intervention strategies to improve health among other veterans and the millions of individuals with PTSD more broadly.

These results have important implications for clinical practice for the treatment of PTSD and prevention of ill health, both broadly and among veterans specifically. Most broadly, our findings combined with past studies [32–35, 55, 56] suggest that people with current PTSD are aging at a faster rate than those without PTSD. Notably, we found that veterans who recovered from PTSD were aging at a rate more similar to those who never met criteria for PTSD, suggesting there may be some degree of reversibility in accelerated aging among individuals with PTSD. However, it is also possible people who are aging more slowly also recover more readily from PTSD and future longitudinal work is still needed to test the reversibility hypothesis. People with faster aging develop more chronic diseases, have higher rates of disability, and greater risk of premature mortality [23, 24]. Future studies should examine whether efficacious PTSD treatments can slow the rate at which people with PTSD are biologically aging [25, 26, 29], in line with a prior randomized control trial of caloric restriction [57]. The ability to measure epigenetic biological aging using DNAm methods provides a promising surrogate clinical outcome that can be assessed before and after treatment with relevance to future health.

The results of this study also have clinical relevance to populations at the greatest risk of developing PTSD, particularly military veterans. An increasing proportion of the VA population are comprised of the large cohorts of Gulf War and post-9/11 deployment veterans, with average ages of 50 and 37 years, respectively [58]. These two eras now account for approximately half the U.S. veteran population [59] and this cohort will require increasing levels of medical care as they age. It is generally more efficacious and cost-effective to prevent ill health from occurring compared to treating chronic diseases after they have developed [29]. Slowing the rate at which younger cohorts of veterans are aging by treating PTSD, a common mental health condition experienced by those cohorts, would have immense public health and economic value, in addition to the improved health and well-being of individuals receiving treatment. These realities combined with our findings offer a time-sensitive opportunity to leverage the reach of integrated medical systems, such as the U.S. Veterans Health Administration, to test the efficacy and effectiveness of slowing aging by treating PTSD and health-relevant sequelae of PTSD.

This study has specific strengths, including the size of the sample, the inclusion of two self-reported racial/ethnic groups, a sizable number of women veterans, and multiple methods of PTSD assessment. Our findings for the associations of trauma, PTSD, and biological aging were conducted in a sample that included two major racial/ethnic groups—non-Hispanic Black people and non-Hispanic White people. Our sample also included almost 500 women, which reflects the changing demographics among the U.S. military veteran population. Our secondary analyses suggest that the association of PTSD and DunedinPACE might be stronger among non-Hispanic White veterans, whereas the association between trauma burden and DunedinPACE might be stronger among women. Future studies would benefit from further investigating the association between these demographic characteristics and DunedinPACE to better characterize which post-9/11 veterans might benefit the most from efficacious PTSD treatment in order to slow aging. Our study also included multiple measures of PTSD (clinical interview, self-report), a comprehensive assessment of trauma [39], and was conducted in a veteran sample with a high prevalence of PTSD. The size and diversity of the sample, the use of multiple assessment modalities, and the relatively high prevalence of PTSD are strengths that provide converging evidence as to the reliability and generalizability of the link between PTSD and accelerated biological aging in a well-powered study. More empirical work is still needed to test the behavioral and psychosocial sequelae of PTSD in additional racial and ethnic groups and among additional non-military samples. However, biological aging will likely be useful as an inclusive physiological biomarker that can be used as a surrogate endpoint in future research studying the causal pathways from PTSD to clinical health outcomes [9–11], complementing disease-specific or system-specific perspectives.

The results of the current study should be understood in the context of its limitations. First, the study was conducted among U.S. military veterans, which may limit the ability to generalize to civilian populations [32]. Though the characteristics of the cohort as post-9/11 veterans provides important benefits in terms of clinical application, this may limit the ability to generalize these results to other veteran cohorts. Second, trauma, PTSD, and DNAm were assessed at a single timepoint. Future studies would benefit from examining PTSD and biological aging longitudinally, which would provide insight into the temporal ordering of these associations and test change DunedinPACE among people whose PTSD goes into remission. Third, the study was correlational and cannot be used to determine causal inferences for the associations of interest. Experimental designs, such randomized control trials, would be needed to causally link PTSD and accelerated aging. Fourth, the current study assessed PTSD using the most current (at the time of assessment) DSM-IV criteria for diagnosis. It is unclear whether similar associations would emerge with DSM-5 criteria. Finally, biological aging was assessed using an epigenetic aging measure applied to participants’ methylation. Although DunedinPACE has been validated in a number of prior studies and external cohorts [27] and was selected due to being trained directly on a biomarker-derived measure of aging, it remains an indirect measure of biological aging and other biological aging measures, such as epigenetic clocks, might show different associations. The measure was created in a non-military New Zealander sample, and future research will be needed to determine to what extent different DunedinPACE aging score correspond to different clinical outcomes.

## Conclusions

In a cohort of 2309 veterans assessed for trauma exposure and PTSD, veterans with current PTSD showed accelerated rates of biological aging. Veterans who had a history of PTSD did not evidence accelerated aging compared to those with current PTSD. The results suggest trauma and PTSD may accelerate biological aging, which could help explain the increased risk for poor health observed among people with PTSD. In addition, the results highlight the importance of determining whether PTSD treatment might slow biological aging and improve health, particularly among groups that are at increased risk of PTSD, such as veterans.

### Supplementary information

Supplemental Material

## Data Availability

Data from the Post Deployment Mental Health Study is part of a Veterans Affairs data repository and is available to researchers who request access through the VISN 6 MIRECC and follow the appropriate data access protocols.
