# Supplementary material for: Posttraumatic stress disorder, trauma, and accelerated biological aging among post-9/11 veterans
Source: Transl Psychiatry. 2024 Jan 6;14:4. doi: 10.1038/s41398-023-02704-y (PMC10771513; doi:10.1038/s41398-023-02704-y)
Supplement: Supplementary file 1 — Supplemental Material [file 41398_2023_2704_MOESM1_ESM.docx]

**Posttraumatic stress disorder, trauma, and accelerated biological aging among post-9/11 Veterans**

Kyle J. Bourassa et al.

**Supplemental Materials**

**Supplemental Text 1.** ….…………….…………….………………………….….………………………………….……………...………2

**Supplemental Analyses 1.** ……………………….………………………….….………………………………….……………...……….3

**Supplemental Table 1.** …………………………….………………………….….………………………………….……………...……….4

**Supplemental Table 2.** …………………………….………………………….….………………………………….……………...……….5

**Supplemental Table 3.** …………………………….………………………….….………………………………….……………...……….6

**Supplemental Analyses 2.** ……………………….………………………….….………………………………….……………...……….7

**Supplemental Table 4.** …………………………….………………………….….………………………………….……………...……….8

**Supplemental References.** …………………………….…………………….….………………………………….……………...………9

**Supplemental Text 1.** **Description of DunedinPACE and Pace of Aging Background.** DunedinPACE was developed using a deeply-phenotyped cohort—the Dunedin study—assessed from birth to age 45. First, participant’s biological aging was measured using the Pace of Aging (1-2), a measure derived from repeated assessments of a panel of 19 biomarkers at ages 26, 32, 38, and 45. Linear change associated with each biomarker for each participant was assessed using mixed modeling; the 19 resulting slopes were then summed to measure decline in physiological functioning across 2 decades. Second, a DNAm measure of the Pace of Aging was created that could be exported to other datasets was derived using elastic net regression and limited to reliable CpG probes (42) to produce DunedinPACE, which has been validated in numerous external datasets (27). To calculate DunedinPACE values for participants in the current study, we applied code provided in the original DunedinPACE publication (38), which uses data from 20,000 probes (173 comprising DunedinPACE and 19,827 randomly selected additional probes) quantile-normalized to match the distribution of the reference Dunedin Study sample. This produced an aging score for each veteran in which a value of one represents 1 year of biological aging per chronological year (i.e. expected aging), with higher scores representing faster aging

**Supplemental Analyses 1.** **Controlling for Cell Type.** We conducted additional analyses that included cell count proportions for the major types of white blood cells (T lymphocytes (CD4+ and CD8+), B cells (CD19+), monocytes (CD14+), NK cells (CD56+) and Neutrophils). The FlowSorted.Blood.450k and FlowSorted.Blood.EPIC packages were used to derive values for the Illumina IlluminaHumanMethylation450 and HumanMethylationEPIC (EPIC)) DNA methylation microarrays, respectively (4). These values were then included as covariates in the primary study models to ensure cell count did not explain the observed associations. As shown in Supplemental Table 1, all main study findings replicated across models assessing PTSD and trauma.

**Supplemental Table 1.** *Primary Study Results of PTSD and Trauma with* *DunedinPACE while Controlling for Cell Type Proportions*

|  | Age-adjusted Bivariate | | Adding demographics | | Adding smoking status | |
| --- | --- | --- | --- | --- | --- | --- |
| *N* = 2,309 | β | 95% CI | β | 95% CI | β | 95% CI |
| Current PTSD | 0.16** | [0.09, 0.24] | 0.19** | [0.12, 0.26] | 0.12** | [0.05, 0.19] |
| PTSD symptoms | 0.10** | [0.06, 0.13] | 0.12** | [0.08, 0.15] | 0.07** | [0.04, 0.11] |
| Lifetime trauma burden | 0.09** | [0.06, 0.13] | 0.08** | [0.05, 0.12] | 0.05** | [0.02, 0.08] |
| *Note:* Current PTSD indicates participants with current PTSD or no current PTSD, PTSD symptoms measures self-reported PTSD symptoms, lifetime trauma burden assesses count of trauma categories experienced across the lifespan. Each model adds more covariates to the model. CI = confidence interval.  * *p* < .05. ** *p* < .01. | | | | | | |

**Supplemental Table 2.** *Association of Aging with PTSD Measures among Post-9/11 Veterans*

|  | Age-adjusted Bivariate | | Adding demographics | | Adding smoking status | |
| --- | --- | --- | --- | --- | --- | --- |
| *N* = 2,309 | β | 95% CI | β | 95% CI | β | 95% CI |
| Clinical interview PTSD | 0.19** | [0.11, 0.28] | 0.18** | [0.10, 0.27] | 0.10* | [0.01, 0.08] |
| Age |  |  | 0.24** | [0.20, 0.28] | 0.27** | [0.23, 0.30] |
| Gender |  |  | 0.30** | [0.21, 0.40] | 0.33** | [0.24, 0.42] |
| Race/ethnicity |  |  | -0.31** | [-0.37, -0.23] | -0.38** | [-0.45, -0.30] |
| Education |  |  | -0.07** | [-0.11, -0.03] | -0.04* | [-0.07, -0.00] |
| Methylation chip |  |  | -0.11** | [-0.21, -0.01] | -0.15** | [-0.24, -0.06] |
| Smoking Status |  |  |  |  | 0.33** | [0.29, 0.36] |
|  |  |  |  |  |  |  |
| Self-reported PTSD | 0.24** | [0.16, 0.32] | 0.22** | [0.14, 0.30] | 0.15** | [0.07, 0.23] |
| Age |  |  | 0.24** | [0.20, 0.28] | 0.27** | [0.23, 0.31] |
| Gender |  |  | 0.31** | [0.22, 0.41] | 0.34** | [0.25, 0.43] |
| Race/ethnicity |  |  | -0.29** | [-0.36, -0.21] | -0.37** | [-0.44, -0.29] |
| Education |  |  | -0.06** | [-0.10, -0.02] | -0.04 | [-0.08, 0.00] |
| Methylation chip |  |  | -0.13** | [-0.23, -0.03] | -0.15** | [-0.24, -0.06] |
| Smoking Status |  |  |  |  | 0.33** | [0.29, 0.36] |
| *Note:* Clinical interview PTSD was assessed using the DSM-IV whereas self-reported PTSD was assessed using Davidson Trauma Scale scores, with scores of 35 or above coded as current PTSD. Each model adds more covariates to the model. PTSD is coded 0 = no PTSD, 1 = current PTSD; gender is coded 0 = men, 1 = women; race/ethnicity is coded 0 = non-Hispanic Black, 1 = non-Hispanic White; methylation chip is coded 0 = Infinium HumanMethylation450 BeadChip, 1 = Infinium MethylationEPIC BeadChip, smoking status is coded 0 = never smoked, 1 = past smoking, 2 = current smoking. CI = confidence interval.  * *p* < .05. ** *p* < .01. | | | | | | |

**Supplemental Table 3.** *Primary Study Results while Stratifying by Self-Reported Race/ethnicity and Gender*

|  | Age-adjusted Bivariate | | Adding demographics | | Adding smoking status | |
| --- | --- | --- | --- | --- | --- | --- |
|  | β | 95% CI | β | 95% CI | β | 95% CI |
| **Non-Hispanic Black Veterans (*n* = 1,109)** | | |  |  |  |  |
| Current PTSD | 0.10 | [-0.02, 0.21] | 0.11 | [-0.01, 0.21] | 0.05 | [-0.05, 0.16] |
| PTSD symptoms | 0.06* | [0.01, 0.12] | 0.07* | [0.01, 0.13] | 0.03 | [-0.02, 0.09] |
| Lifetime trauma burden | 0.04 | [-0.02, 0.10] | 0.05 | [-0.00, 0.11] | 0.03 | [-0.02, 0.09] |
|  |  |  |  |  |  |  |
| **Non-Hispanic White Veterans (*n* = 1,200)** | | |  |  |  |  |
| Current PTSD | 0.34** | [0.24, 0.46] | 0.32** | [0.22, 0.42] | 0.22** | [0.12, 0.32] |
| PTSD symptoms | 0.20** | [0.14, 0.25] | 0.19** | [0.13, 0.24] | 0.12** | [0.07, 0.18] |
| Lifetime trauma burden | 0.13** | [0.08, 0.19] | 0.12** | [0.07, 0.18] | 0.07** | [0.02, 0.13] |
|  |  |  |  |  |  |  |
| **Female Veterans (*n* = 491)** | | |  |  |  |  |
| Current PTSD | 0.29** | [0.12, 0.46] | 0.29** | [0.12, 0.46] | 0.23** | [0.06, 0.40] |
| PTSD symptoms | 0.15** | [0.07, 0.24] | 0.15** | [0.07, 0.24] | 0.12** | [0.04, 0.20] |
| Lifetime trauma burden | 0.13** | [0.04, 0.22] | 0.18** | [0.09, 0.26] | 0.15** | [0.06, 0.23] |
|  |  |  |  |  |  |  |
| **Male Veterans (*n* = 1,818)** | | |  |  |  |  |
| Current PTSD | 0.23** | [0.07, 0.16] | 0.20** | [0.11, 0.29] | 0.12** | [0.04, 0.20] |
| PTSD symptoms | 0.14** | [0.09, 0.18] | 0.12** | [0.08, 0.17] | 0.08** | [0.04, 0.11] |
| Lifetime trauma burden | 0.07** | [0.06, 0.14] | 0.07** | [0.02, 0.11] | 0.03 | [-0.01, 0.07] |
| *Note:* Current PTSD indicates participants with current PTSD or no current PTSD, PTSD symptoms measures self-reported PTSD symptoms, lifetime trauma burden assesses count of trauma categories experienced across the lifespan. Each model adds more covariates to the model. CI = confidence interval.  * *p* < .05. ** *p* < .01. | | | | | | |

**Supplemental Analyses 2. Primary study results when controlling from smoking methylation score.** We conducted additional analyses that accounted for smoking methylation scores rather than self-reported smoking status. We used an established methylation measure of smoking that was derived from smoking history (5-6) to calculate smoking methylation scores for the study sample. Methylation scores correlated with our smoking measure (*r* = .56, p < .001). All primary study findings replicated while including this smoking methylation score as a covariate in the primary study models. While controlling for smoking methylation scores (in addition to all other covariates), veterans with current PTSD were aging faster biologically compared to veterans without PTSD, β = 0.13, 95% CI [0.05, 0.14], *p* < .001, veterans who reported more PTSD symptoms also had faster DunedinPACE compared to veterans reporting fewer PTSD symptoms, β = 0.07, 95% CI [0.03, 0.11], *p* < .001, veterans who reported more trauma exposure showed faster DunedinPACE compared to veterans with less trauma exposure, β = 0.06, 95% CI [0.02, 0.09], *p* = .002, and veterans with past PTSD continued to evidence slower DunedinPACE than those with current PTSD, β = -0.17, 95% CI [-0.30, -0.04], *p* = .013.

**Supplemental References**

1. Belsky DW, Caspi A, Houts R, Cohen HJ, Corcoran DL, Danese A, et al. Quantification of biological aging in young adults. *Proc Natl Acad Sci*. 2015; 112(30): E4104-10. doi:10.1073/pnas.1506264112.
2. Elliott ML, Caspi A, Houts RM, Ambler A, Broadbent JM, Hancox RJ, et al. Disparities in the pace of biological aging among midlife adults of the same chronological age have implications for future frailty risk and policy. *Nat Aging*. 2021;1(3):295-308. doi: 10.1038/s43587-021-00044-4.
3. Sugden K, Hannon EJ, Arseneault L, Belsky DW, Corcoran DL, Fisher HL, et al. Patterns of Reliability: Assessing the Reproducibility and Integrity of DNA Methylation Measurement. *Patterns*. 2020 8;1(2):100014. doi:10.1016/j.patter.2020.100014
4. Houseman EA, Accomando WP, Koestler DC, Christensen BC, Marsit CJ, Nelson HH, et al. DNA methylation arrays as surrogate measures of cell mixture distribution. *BMC Bioinformat*. 2012;13(1):1-6.
5. Sugden K, Hannon EJ, Arseneault L, Belsky DW, Broadbent JM, Corcoran DL, et al. Establishing a generalized polyepigenetic biomarker for tobacco smoking*. Transl Psychiat*. 2019;9(1):92.
6. Joehanes R, Just AC, Marioni RE, Pilling LC, Reynolds LM, Mandaviya PR, et al. Epigenetic signatures of cigarette smoking. *Circ. Cardiovasc Genet.* 2016;9:436–447.
